# Early skeletal muscle pathology and disease progress in the *dy*^*3K*^*/dy*^*3K*^ mouse model of congenital muscular dystrophy with laminin α2 chain-deficiency

**DOI:** 10.1038/s41598-019-50550-0

**Published:** 2019-10-04

**Authors:** Kinga I. Gawlik, Zandra Körner, Bruno M. Oliveira, Madeleine Durbeej

**Affiliations:** Muscle Biology Unit, Department of Experimental Medical Science, Lund University, Lund, Sweden

**Keywords:** Mechanisms of disease, Experimental models of disease

## Abstract

Deficiency of laminin α2 chain leads to a severe form of congenital muscular dystrophy (LAMA2-CMD), and dystrophic symptoms progress rapidly in early childhood. Currently, there is no treatment for this detrimental disorder. Development of therapies is largely hindered by lack of understanding of mechanisms involved in the disease initiation and progress, both in patients but also in mouse models that are commonly used in the preclinical setup. Here, we unveil the first pathogenic events and characterise the disease development in a mouse model for LAMA2-CMD (*dy*^*3K*^*/dy*^*3K*^), by analysing muscles at perinatal, neonatal and postnatal stages. We found that apoptotic muscle fibres were present as early as postnatal day 1. Other typical dystrophic hallmarks (muscle degeneration, inflammation, and extensive production of the extracellular matrix proteins) were clearly evident already at postnatal day 4, and the highest degree of muscle deterioration was reached by day 7. Interestingly, the severe phenotype of limb muscles partially recovered on days 14 and 21, despite worsening of the general condition of the *dy*^*3K*^*/dy*^*3K*^ mouse by that age. We found that masticatory muscles were severely affected in *dy*^*3K*^*/dy*^*3K*^ mice and this may be an underlying cause of their malnutrition, which contributes to death around day 21. We also showed that several signalling pathways were affected already in 1-day-old *dy*^*3K*^*/dy*^*3K*^ muscle. Therapeutic tests in the *dy*^*3K*^*/dy*^*3K*^ mouse model should therefore be initiated shortly after birth, but should also take into account timing and correlation between regenerative and pathogenic events.

## Introduction

Muscular dystrophy is a heterogonous group of incurable inherited diseases mainly affecting skeletal muscle. Congenital muscular dystrophy type 1A (LAMA2-CMD) represents one of the most common and one of the most severe disorders among the congenital muscular dystrophy subgroup^1^, arising from mutations in the gene encoding laminin α2 chain^2^. This extracellular matrix protein, a constituent of the laminin-211 heterotrimer, provides stability to muscle fibres and regulates their homeostasis^3,4^. The majority of individuals suffering from LAMA2-CMD with complete laminin α2 chain-deficiency display muscle weakness and hypotonia immediately after birth. Subsequently, severe muscle wasting, joint contractures, gravely impaired motor function and respiratory difficulties dramatically impact the condition of young patients. In many cases, premature death is a consequence of these progressive symptoms^1,2,5^. Typical hallmarks of LAMA2-CMD dystrophic muscle biopsy include: degenerating/regenerating muscle fibres, muscle fibre atrophy, necrosis and apoptosis of muscle cells, early inflammation and extensive connective tissue infiltration^5–8^.

Deeper understanding of mechanisms triggering the disease initiation, early pathology and progress is essential for designing future treatment strategies. Mouse models for laminin α2 chain-deficiency serve as a tool for investigating biological and molecular interactions underlying LAMA2-CMD pathogenesis^6^. Two studies have described the early disease signature in *dy*^*W*^*/dy*^*W*^ embryos and in 1–4-week-old *dy*^*W*^*/dy*^*W*^ mice^9,10^ (these animals display a fairly severe phenotype and express low amounts of truncated laminin α2 subunit), but our knowledge about early events in laminin α2 chain-deficient dystrophic muscle is still insufficient.

Very little is known about disease development in *dy*^*3K*^*/dy*^*3K*^ mice, representing the only mouse model with complete loss of laminin α2 chain that displays the most severe phenotype among LAMA2-CMD murine mutants. Various therapeutic approaches targeting pathogenic mechanisms in the *dy*^*3K*^*/dy*^*3K*^ mouse were instigated at 2–3 weeks of age, and it is reasonable to assume that an earlier intervention would yield better treatment outcome. However, targeting certain pathological processes (e.g. inflammation) must be coordinated accordingly throughout the disease progress, in order to circumvent the disruption of muscle repair interactions. For optimal design of preclinical studies aimed at preventing the disease in *dy*^*3K*^*/dy*^*3K*^ animals, it is crucial to characterise the timing of pathology hallmarks in a wide range of laminin α2-chain-deficient muscles.

## Results

### General phenotype and muscle function throughout postnatal development of *dy*^*3K*^*/dy*^*3K*^ mice

**Figure 1:**
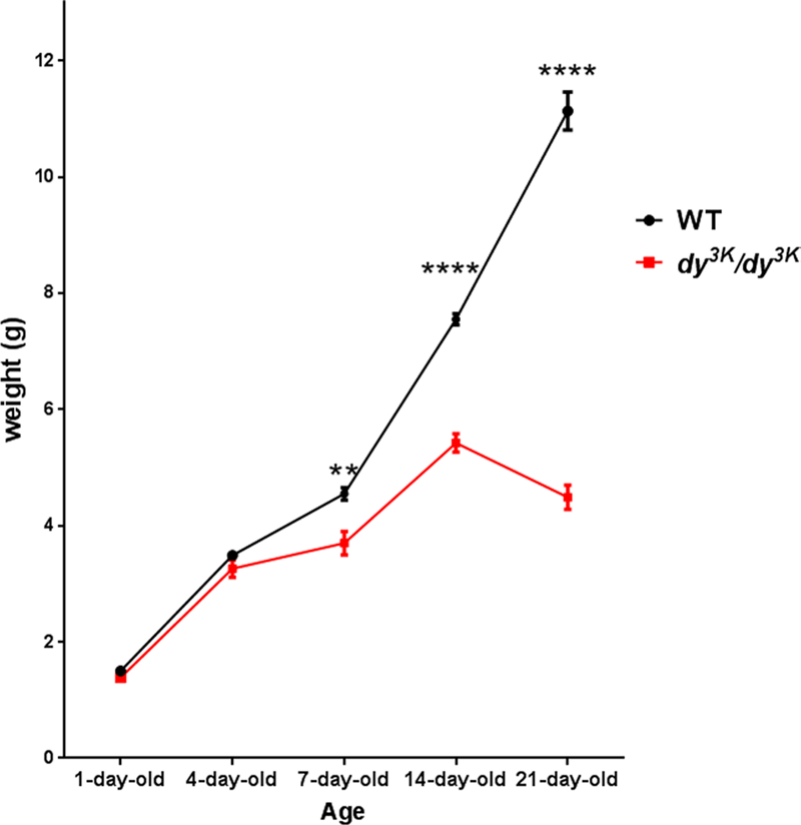
Weight analysis of *dy*^*3K*^*/dy*^*3K*^ mice over the course of the disease. Wild-type and *dy*^*3K*^*/dy*^*3K*^ mice were weighed at the age of 1, 4, 7, 14 and 21 days (for day 1: n = 17, n = 7, respectively; for day 4: n = 25, n = 5, respectively; for day 7: n = 30, n = 6, respectively; for day 14: n = 8, n = 8, respectively, for day 21: n = 6, n = 8, respectively). Significant difference in weight between sick and healthy mice is marked at day 7 (t-test, p = 0.0025) and the weight disparity becomes even more evident with age (day 14 and 21, t-test, p < 0.0001).

Two-week-old *dy*^*3K*^*/dy*^*3K*^ pups can be clearly identified due to their smaller size^11–13^, but younger mice have not been thoroughly analysed previously. We compared *dy*^*3K*^*/dy*^*3K*^ and wild-type body weights at postnatal day 1, 4, 7, 14 and 21. No significant difference between the groups was noted in 1- and 4-day-old mice, but the weight gain delay in *dy*^*3K*^*/dy*^*3K*^ mice was evident at day 7. The disparity between the weights of wild-type and dystrophic animals further increased on days 14 and 21. Between these two time points, *dy*^*3K*^*/dy*^*3K*^ mice started losing their already low weight (Fig. 1). It is important to mention that over the years the phenotype of *dy*^*3K*^*/dy*^*3K*^ mice in our colony has gradually worsened. The terminal stage of the disease was between day 28–35^14,15^, whereas currently *dy*^*3K*^*/dy*^*3K*^ animals do not survive longer than 21 days (data not shown).

**Figure 2:**
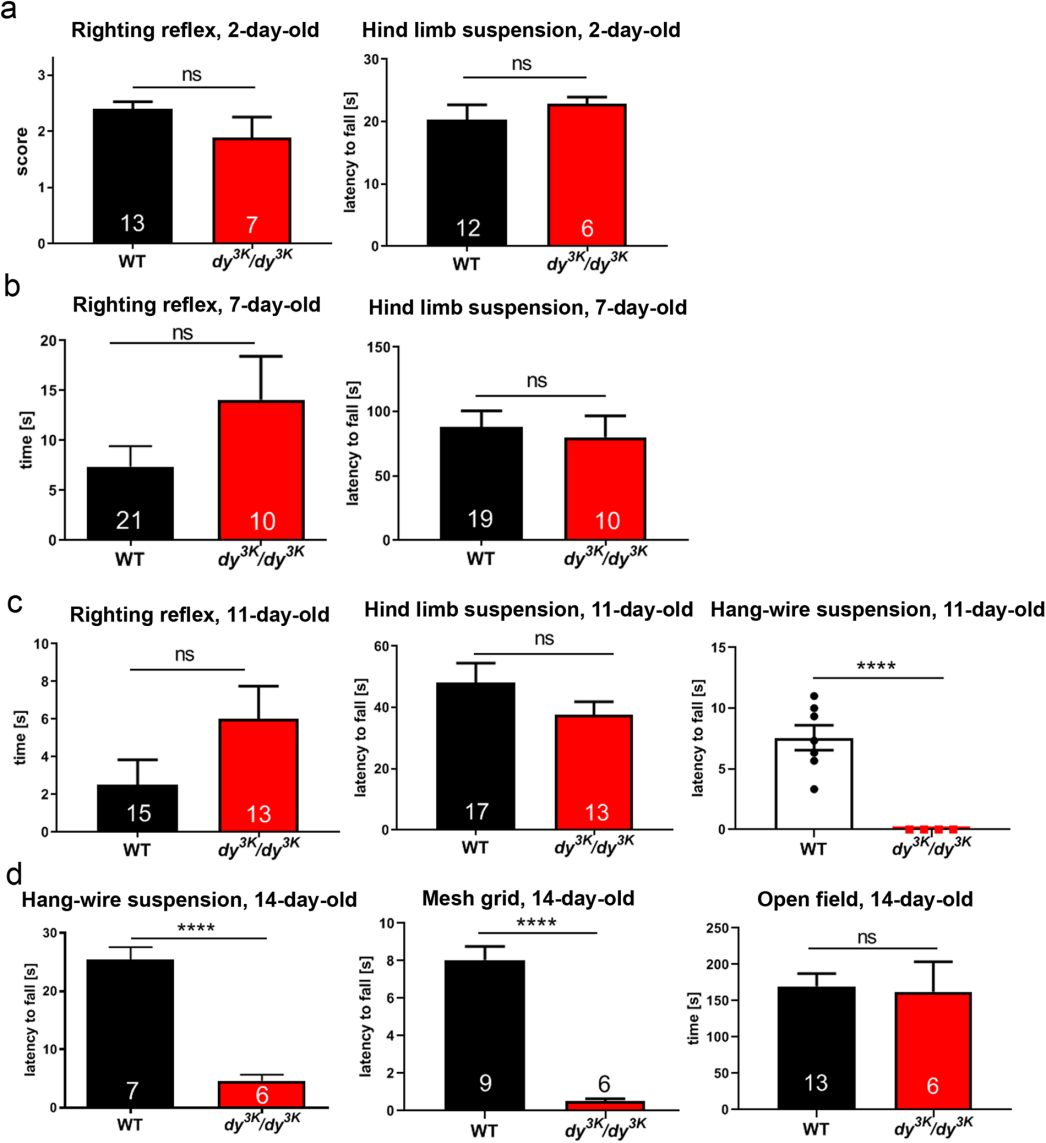
Muscle function in *dy*^*3K*^*/dy*^*3K*^ mice throughout the disease course. (**a,b**) Righting reflex and hind limb suspension test do not show significant impairment of neuromuscular function in 2- and 7-day-old *dy*^*3K*^*/dy*^*3K*^ pups. Righting reflex in 2-day-old mice was scored as follows: 0: no attempt to right itself, very passive; 1: poor attempt to right itself; 2: fair attempt to right itself, active; 3: managed to turn. (**c**) Righting reflex, hind limb suspension and grip strength (hang-wire) test in 11-day-old animals. No striking difficulties in performance of *dy*^*3K*^*/dy*^*3K*^ mice were noted during righting reflex and hind limb suspension tests. Hang-wire test showed dysfunction of *dy*^*3K*^*/dy*^*3K*^ neuromuscular system when attempting a more complex physical activity. (**d**) Two grip strength tests (hang-wire and mesh grid) and open field test (activity in a new cage) in 14-day-old mice. Both grip strength tests show enormous impairment of muscle function in *dy*^*3K*^*/dy*^*3K*^ mice. The performance of *dy*^*3K*^*/dy*^*3K*^ animals in the open field test, on the other hand, was not significantly diminished.

Muscle function is compromised in laminin α2 chain-deficiency, and it represents a critical outcome measure in studying dystrophic phenotypes. We have demonstrated before that 21-day-old *dy*^*3K*^*/dy*^*3K*^ mice are less active and weaker^11,12,16–18^ (and data not shown). Using a battery of functional tests, we have now tested when muscle function impairment becomes evident in *dy*^*3K*^*/dy*^*3K*^ animals. We subjected healthy controls and *dy*^*3K*^*/dy*^*3K*^ individuals (2-, 7- and 11-day-old) to righting reflex and hind limb suspension tests. The righting reflex evaluates general body strength and can be affected by weakness in limb and trunk muscles^19,20^. The suspension test assesses hind limb muscle strength, fatigue and general neuromuscular function^21^. For both tests two types of measurements were considered: time and score. Neither righting reflex (score) nor capacity to support the body when suspended on hind limbs (time and score) were compromised in 2-day-old *dy*^*3K*^*/dy*^*3K*^ mice (Fig. 2a and Supplementary Fig. 1a). Seven-day-old *dy*^*3K*^*/dy*^*3K*^ animals did not show significant motor impairments either, and all of them were able to right themselves very quickly (all score 3, data not shown). The only visible struggle was a tendency to fail to pull one paw (most often a hind limb) from underneath the trunk and spread it correctly on the bench (slightly reflected in time measurement, when this feature was taken into consideration) (Fig. 2b and Supplementary Fig. 1b). Despite looking weaker and fragile, 7-day-old *dy*^*3K*^*/dy*^*3K*^ animals were able to hold onto the rim of the 50 ml tube during the hind limb suspension test, and did not display hind leg clasping that is characteristic for mice with impaired neuromuscular function (Fig. 2b and Supplementary Fig. 1b).

The righting reflex had a similar characteristic in 11-day-old *dy*^*3K*^*/dy*^*3K*^ mice; in general they swiftly returned from a supine position to their paws, only slightly slower than wild-type mice (Supplementary Fig. 1b, Fig. 2c). They struggled sometimes with placing one limb in a stretched position (Fig. 2c). Eleven-day-old *dy*^*3K*^*/dy*^*3K*^ mice also looked weaker when performing the hind limb suspension test, but again this experiment did not show significant differences between dystrophic animals and healthy controls (Fig. 2c), maybe because wild-type mice often chose to fall down despite not having troubles with holding onto the tube rim. A tendency for increased hind limb clasping during this experiment (score, Fig. 2c) and during the tail suspension test (data not shown) was visible in *dy*^*3K*^*/dy*^*3K*^ mice at this age. A more challenging functional test (hang-wire suspension) revealed that the 11-day-old *dy*^*3K*^*/dy*^*3K*^ mice are indeed significantly weaker than healthy littermates (Fig. 2c). This test examines forelimb strength^20,22^, but also general fitness and coordination. Wild-type mice did not support their body on the wire for very long, but *dy*^*3K*^*/dy*^*3K*^ mice were virtually unable to perform the test at that age (Fig. 2c). Within only a few days (at the age of 14 days), wild-type animals developed enormous acrobatic skills to support their body on the wire (including not only forelimbs, but also hind limbs) (Fig. 2d, Video 1), but *dy*^*3K*^*/dy*^*3K*^ pups were only occasionally able to hang onto the wire for a few seconds (Fig. 2d, Video 2). The mesh grid test, another challenging test that assesses the strength of all four paws to withstand gravity forces^20,23^, also showed a massive difference between healthy and dystrophic mice at the age of 14 days (Fig. 2d). Finally, we subjected 14-day-old mice to a less demanding open field activity test. Some *dy*^*3K*^*/dy*^*3K*^ mice were moving restlessly, some were inactive and there was no significant difference between the dystrophic and healthy groups (Fig. 2d). The open field activity test might not show motor impairments in a representative way, and more challenging tests are more reliable for assessing motor function in *dy*^*3K*^*/dy*^*3K*^ mice, unless data is acquired through a more sophisticated procedure (for example, distance covered).

### Limb muscle morphology and pathology progression

We have previously demonstrated increased central nucleation of myofibres (a sign of degeneration-regeneration cycle), reduced fibre diameter, augmented apoptosis, increased inflammation and enhanced fibrosis in 14- and 21-day-old (and older) *dy*^*3K*^*/dy*^*3K*^ muscle^12–14,16–18^. In order to characterise the onset and development of those pathological changes, we have now assessed muscles from 18.5-day embryos (E18.5), as well as from 1-, 4-, 7-, 14- and 21-day-old mice (wild-type and *dy*^*3K*^*/dy*^*3K*^).

**Figure 3:**
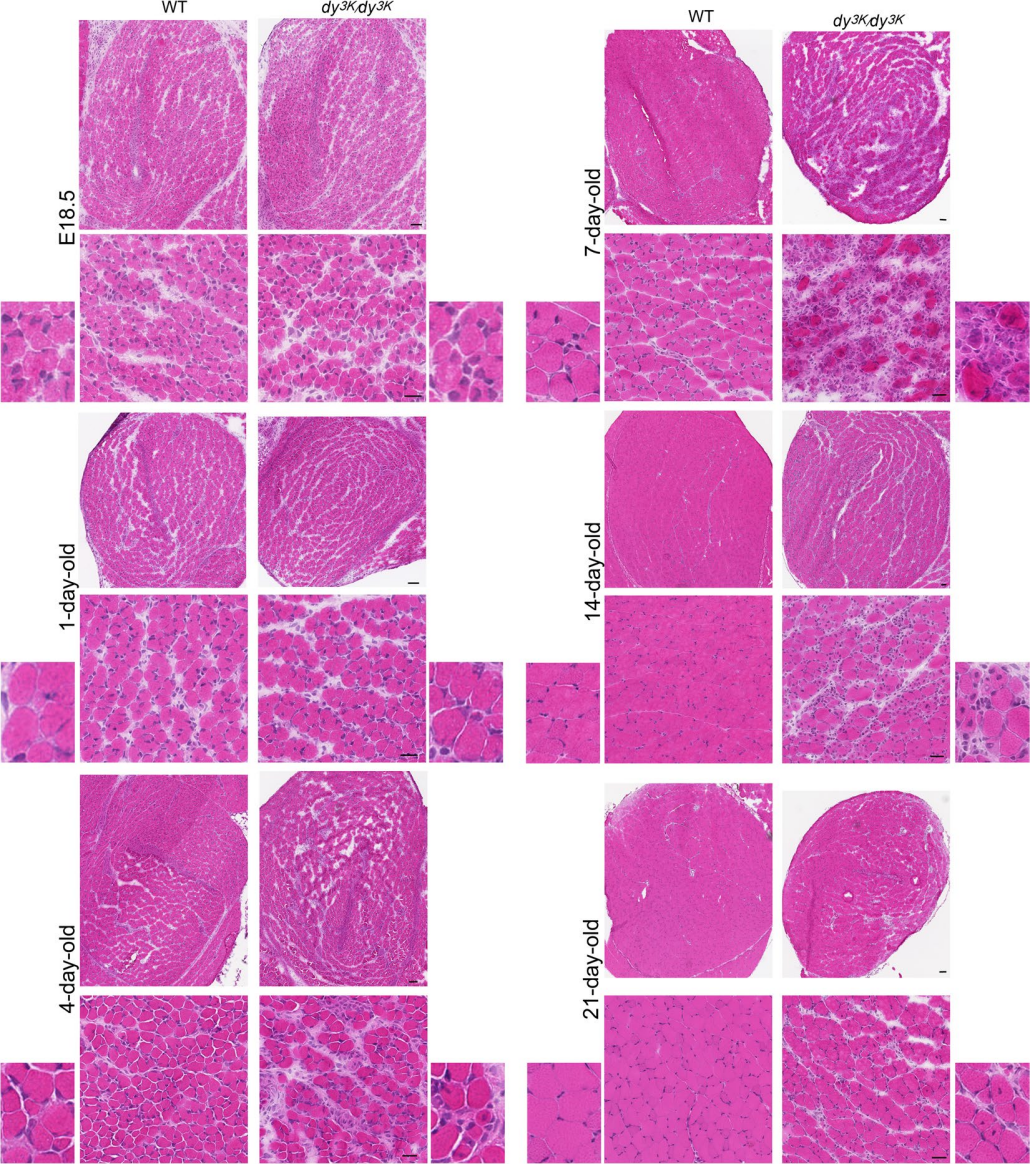
Dystrophic changes in *dy*^*3K*^*/dy*^*3K*^ muscle throughout the disease course (E18.5, postnatal day 1, 4, 7, 14 and 21). Morphology was assessed by haematoxylin & eosin staining. Whole rectus femoris, representative muscle area and magnified panels are shown. Scale bars: for the whole muscle: 50 µm; intermediate magnification panel: 25 µm.

In agreement with analysis of perinatal *dy*^*W*^*/dy*^*W*^ muscles^9^, we did not detect morphology alterations in E18.5 *dy*^*3K*^*/dy*^*3K*^ muscles (rectus femoris, Fig. 3; vastus lateralis and calf muscles, data not shown). Perinatal *dy*^*3K*^*/dy*^*3K*^ muscle showed the same appearance as age-matched wild-type muscle: muscle fibres were clearly formed, yet they were small, loosely packed and often contained centrally located nuclei. One-day-old *dy*^*3K*^*/dy*^*3K*^ muscle also appeared histologically normal, as demonstrated by haematoxylin and eosin staining (rectus femoris, Fig. 3; vastus lateralis and calf muscles, data not shown). A difference in muscle morphology between wild-type and *dy*^*3K*^*/dy*^*3K*^ animals became evident at day 4, when muscle damage and mononuclear cell infiltrates were clearly noticeable (rectus femoris, Fig. 3; vastus lateralis and calf muscles, data not shown). However, the most extreme change in muscle morphology throughout the lifespan of *dy*^*3K*^*/dy*^*3K*^ animals was observed in 7-day-old mice. Loss of muscle fibres, prominent inflammation and deposition of the extracellular matrix at the large areas of muscle were the most striking features (rectus femoris, Fig. 3; vastus lateralis and calf muscles, data not shown). The general appearance of 7-day-old laminin α2 chain-deficient muscle presents the highest degree of overall disruption of muscle fascicles. Strikingly, this dramatic phenotype was fairly well recovered by the age of 14 and 21 days (Fig. 3). The dystrophic features were still present (infiltration of mononuclear cells, centrally nucleated fibres, wider interstitial space), but 14- and especially 21-day-old *dy*^*3K*^*/dy*^*3K*^ muscles showed more compact and healthier appearance of muscle fascicles (Fig. 3) due to regenerative processes. Generally, vastus lateralis was more affected than rectus femoris, and the posterior part of the calf displayed more severe phenotype than anterior muscles (data not shown). Individual differences between *dy*^*3K*^*/dy*^*3K*^ mice from the same age group were observed (extremely affected animals and those displaying slightly milder dystrophic features) (data not shown).

**Figure 4:**
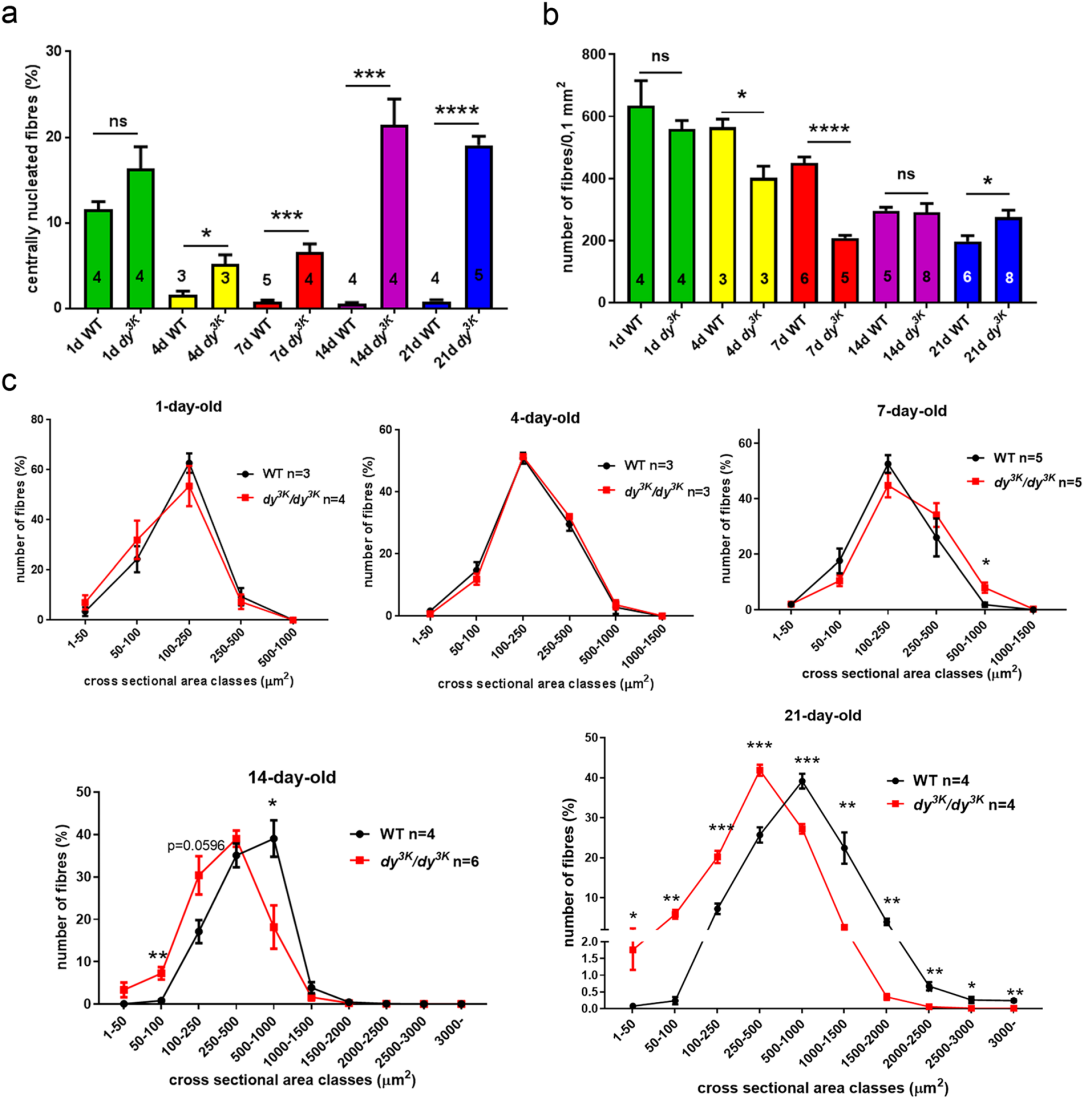
Morphometric analyses of wild-type and *dy*^*3K*^*/dy*^*3K*^ rectus femoris throughout the disease course. **(a)** Quantification of regenerating fibres. The number of centrally nucleated regenerating myofibres is increased in *dy*^*3K*^*/dy*^*3K*^ muscle compared to wild-type muscle from postnatal day 4 onwards (p = 0.0401, p = 0.0004, p = 0.0004, p < 0.0001, for day 4, 7, 14 and 21, respectively). **(b)** The number of muscle fibres per 0.1 mm^2^ (rectus femoris). Muscle fibres are temporarily lost at the age of 4 and 7 days in *dy*^*3K*^*/dy*^*3K*^ rectus femoris (p = 0.002 and p = 0.0024, respectively). **(c)** Analysis of muscle fibre sizes from wild-type and *dy*^*3K*^*/dy*^*3K*^ rectus femoris (day 1, 4, 7, 14 and 21). Muscle atrophy becomes evident in 14-day-old dystrophic muscles, and the shift towards significantly smaller muscle fibres is particularly pronounced at day 21.

Detailed analysis of rectus femoris muscle morphology (quantification of central nucleation, fibre number and fibre size) from wild-type and *dy*^*3K*^*/dy*^*3K*^ animals at different ages confirmed the general histological appearance revealed by haematoxylin and eosin staining. The degeneration/regeneration cycles started at day 4 in *dy*^*3K*^*/dy*^*3K*^ muscle, as demonstrated by increased numbers of centrally nucleated fibres (Fig. 4a). This dystrophic feature was maintained throughout the disease progress, being highly pronounced at day 14 and 21 (Fig. 4a). As a result of muscle damage, the number of muscle fibres decreased significantly at the age of 4 and especially 7 days (Fig. 4b), but fully recovered already in 14-day-old *dy*^*3K*^*/dy*^*3K*^ muscle (Fig. 4b), indicating active regeneration. The amount of muscle fibres even increased in 21-day-old *dy*^*3K*^*/dy*^*3K*^ muscle (Fig. 4b). However, the regeneration process is not entirely efficient/correct in laminin α2 chain deficiency^13,24–27^. Clearly, dystrophic features were not completely reversed despite ongoing muscle repair in *dy*^*3K*^*/dy*^*3K*^ muscle. Most probably the newly formed *dy*^*3K*^*/dy*^*3K*^ myofibres would not reach normal size (see Fig. 4c), because they undergo damage^13^ and cannot grow normally. A secondary loss of muscle fibres would probably occur after the initial wave of muscle repair past an age of 3 weeks, and subsequent regeneration events would not be equally effective. This cannot be established due to death of *dy*^*3K*^*/dy*^*3K*^ animals around day 21. Further studies of muscle regeneration and satellite cells in the *dy*^*3K*^*/dy*^*3K*^ mouse model are necessary.

Muscle atrophy without loss of muscle fibres is a feature of laminin α2 chain-deficiency^11^. By measuring the fibre size, we confirmed that smaller fibres contribute to muscle atrophy in the *dy*^*3K*^*/dy*^*3K*^ mouse model. At the neonatal stage (day 1) and at the initial stage of the muscle pathology (day 4), no shift in the fibre size distribution was detected (Fig. 4c). A tendency for increased fibre size in *dy*^*3K*^*/dy*^*3K*^ muscle at day 7 was revealed (Fig. 4c), probably because bigger fibres remain intact within the *dy*^*3K*^*/dy*^*3K*^ muscle at the onset of massive damage (Fig. 3). At day 14, muscle fibres from *dy*^*3K*^*/dy*^*3K*^ muscle started displaying the shift towards smaller cross-sectional area due to the presence of many small regenerating fibres. This difference became highly significant at day 21 (Fig. 4c). Hence, muscle atrophy in 21-day-old *dy*^*3K*^*/dy*^*3K*^ muscle is due to a shift in the fibre size distribution and not to muscle fibre loss (Fig. 4b,c)^11^.

### Dystrophic processes in *dy*^*3K*^*/dy*^*3K*^ muscle at different stages of the disease

**Figure 5:**
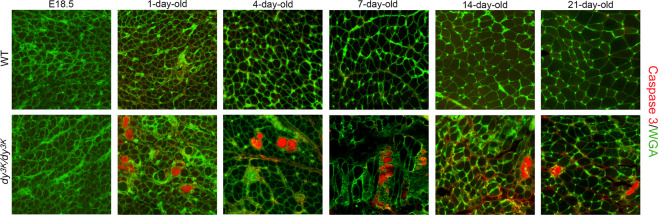
Apoptosis of *dy*^*3K*^*/dy*^*3K*^ muscle fibres. Cell death was determined by caspase-3 immunostaining (red). Wheat germ agglutinin (WGA, in green) was used to visualise muscle fibres. Only E18.5 muscles do not display apoptotic myofibres. Calf and thigh muscles were used. Scale bar: 30 µm.

**Figure 6:**
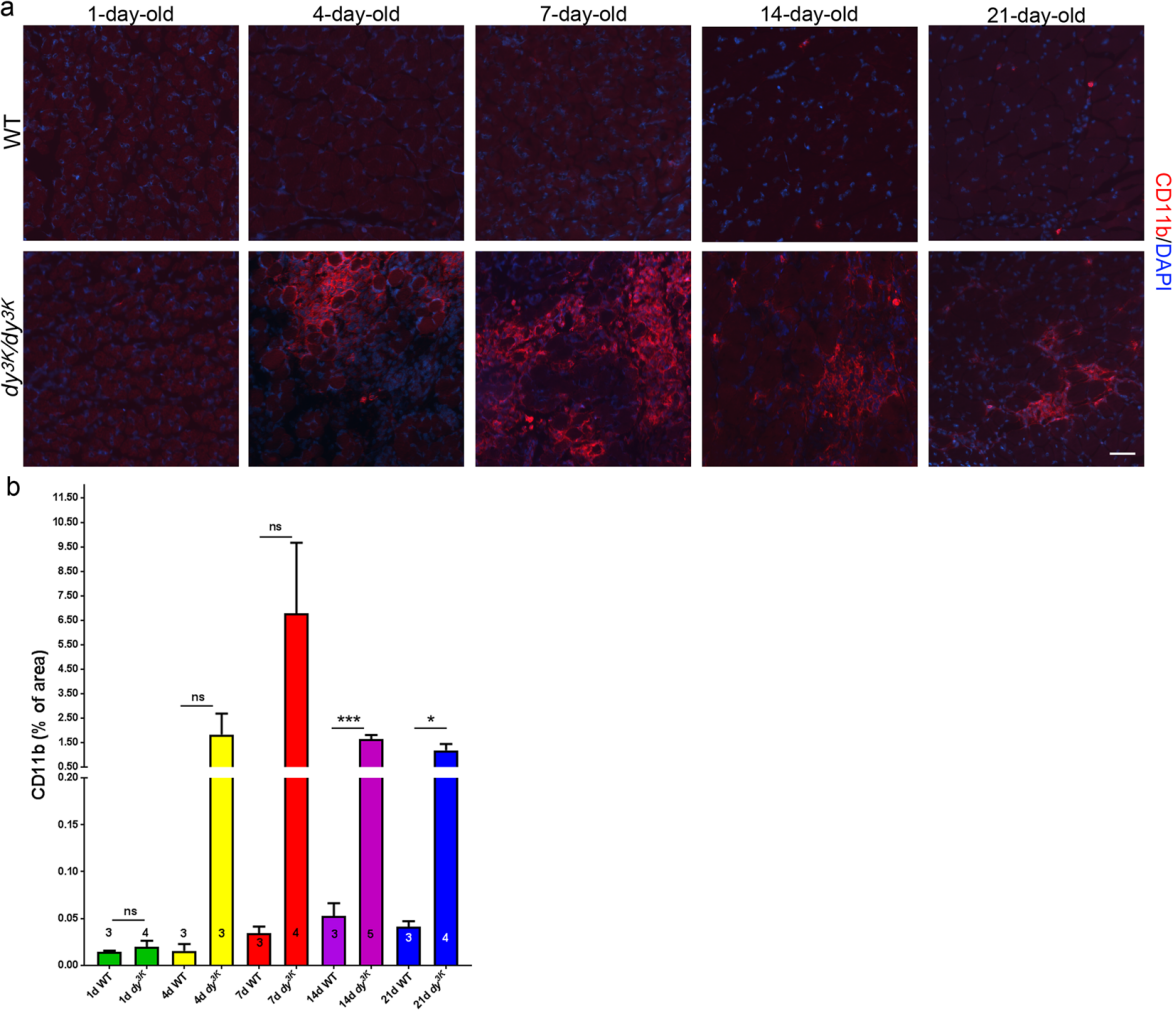
Inflammatory response throughout the disease progression. **(a)** Inflammation was assessed in rectus femoris using CD11b antibody against leukocytes (red). DAPI was used to visualise nuclei. Infiltrates of inflammatory cells become evident at day 4, are highly pronounced at day 7, and decrease afterwards. (**b**) Quantification of CD11b stained areas in wild-type and *dy*^*3K*^*/dy*^*3K*^ muscle. There is a clear trend for increased inflammation in 4- and 7-day-old dystrophic muscle, and the CD11b-positive areas are significantly larger in 14- and 21-day-old *dy*^*3K*^*/dy*^*3K*^ muscle (p = 0.0004 and p = 0.0126, respectively) compared to age-matched wild-type muscle Scale bar: 40 µm.

**Figure 7:**
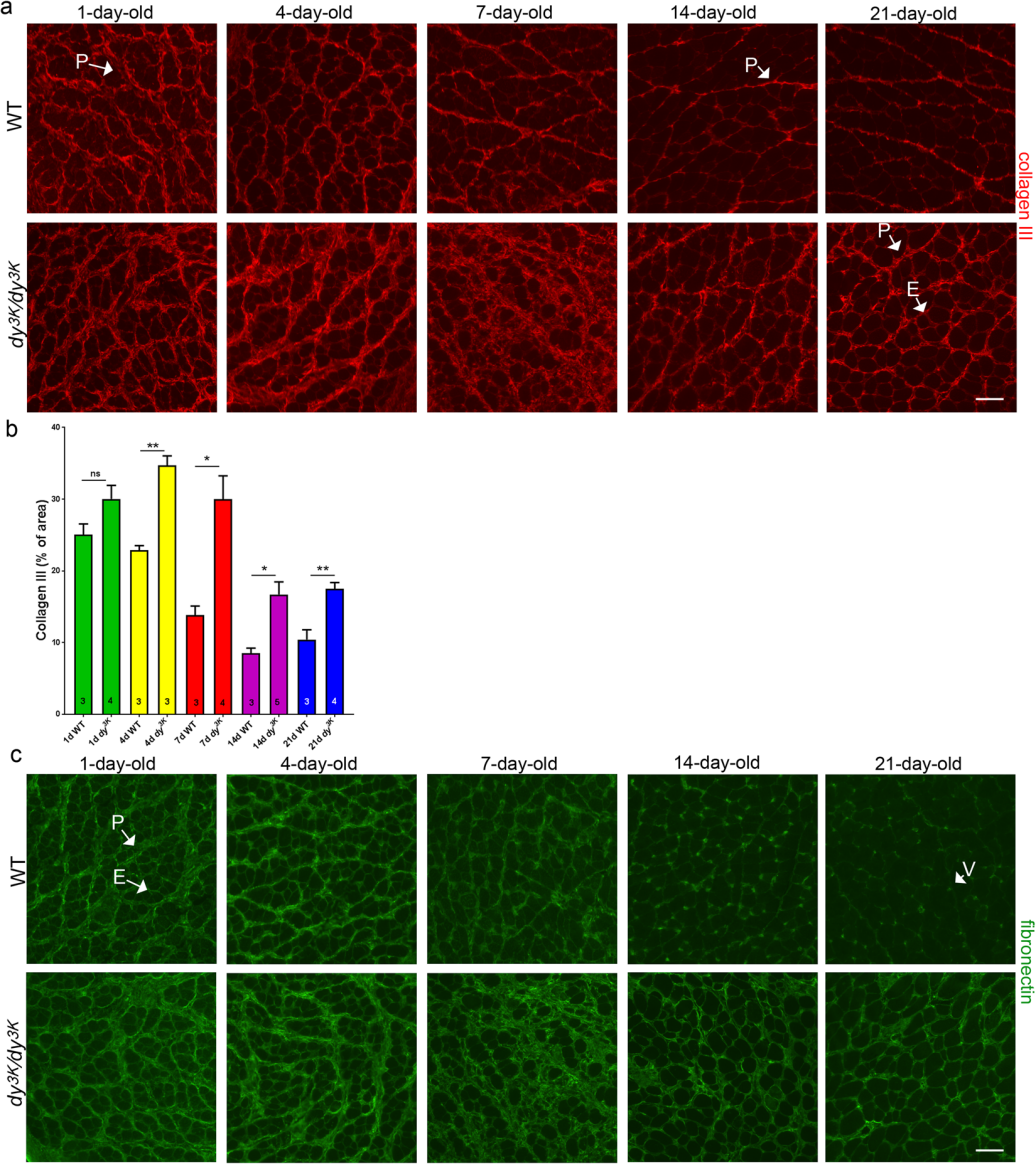
Expression of the extracellular matrix proteins at the different stages of laminin α2 chain-deficient muscular dystrophy. **(a,b)** Collagen III is expressed at similar levels and in the same muscle compartment (perimysium, P) in 1-day-old wild-type and *dy*^*3K*^*/dy*^*3K*^ rectus femoris muscle. Differences in collagen III expression between two analysed groups become evident at day 4 and are especially prominent at day 7: there is a vast increase in collagen III-stained areas in *dy*^*3K*^*/dy*^*3K*^ muscle compared to wild-type control (p = 0.0013 and 0.0096, respectively). Downregulation of collagen III in later stages of the disease (day 14 and 21) was observed, but the deposits of collagen III remain significantly more pronounced than in age-matched wild-type muscle (p = 0.0145 and p = 0.0057, respectively). Collagen III is present both in perimysium (P) and endomysium (E) in *dy*^*3K*^*/dy*^*3K*^ muscle, but it is expressed only in perimysium in wild-type muscle. **(c)** Expression pattern of fibronectin in dystrophic muscle is similar to that observed for collagen III. V-vessel. Scale bars: 40 µm.

We further investigated the specific dystrophic processes in *dy*^*3K*^*/dy*^*3K*^ muscle by immunostaining detecting apoptotic cells (caspase-3, Fig. 5), inflammatory cells (CD11b, Fig. 6; CD68, data not shown) and extracellular matrix deposits (collagen III, fibronectin, Fig. 7). No signs of apoptosis (Fig. 5), inflammation, and no increased content of interstitial extracellular matrix (data not shown) were revealed in *dy*^*3K*^*/dy*^*3K*^ embryonic muscles. Despite no obvious defects in muscle morphology in 1-day-old *dy*^*3K*^*/dy*^*3K*^ muscle (Fig. 3, haematoxylin and eosin staining), apoptotic cells were found at this stage (Fig. 5). Apoptotic events in 1-day-old muscle therefore mark the start of pathology in the laminin α2 chain-deficient *dy*^*3K*^*/dy*^*3K*^ mouse. Apoptosis of muscle fibres was maintained throughout all the stages of the disease (Fig. 5). No other pathological changes (inflammation, increased production of the extracellular matrix proteins) were observed in 1-day-old muscle (Figs 6 and 7).

Muscle pathology advanced rapidly already in 4-day-old *dy*^*3K*^*/dy*^*3K*^ muscle, with clearly visible leukocyte infiltrates that are a consequence of muscle damage (rectus femoris, Fig. 6; vastus lateralis, data not shown, for all ages analysed). The inflammatory response peaked at day 7, and decreased afterwards (day 14 and 21), but was still quite pronounced focally (Fig. 6a). Quantification of the CD11b staining showed a clear trend towards increased presence of leukocytes in the dystrophic muscle at day 4 and 7, and a significant difference between wild-type and age-matched *dy*^*3K*^*/dy*^*3K*^ muscle at day 14 and 21 (Fig. 6b). Similar results were obtained when using the antibody against CD68 (depicting monocytes and macrophages; data not shown).

The expression pattern of the extracellular matrix components (collagen III and fibronectin) in *dy*^*3K*^*/dy*^*3K*^ muscle was similar to the CD11b inflammatory marker: production of collagen III and fibronectin increased at day 4, strongly increased at day 7 and decreased at day 14 and 21 (rectus femoris, Fig. 7a,c; vastus lateralis, data not shown). At all analysed ages, expect for day 1, the expression of collagen III was significantly increased in dystrophic muscle compared to age-matched wild-type muscle, also at the later stages of the disease (day 14, and 21) (Fig. 7b). Notably, visible downregulation of the extracellular matrix proteins occurred in *dy*^*3K*^*/dy*^*3K*^ muscle between day 7 and 14 (Fig. 7a–c). This expression pattern suggests involvement of collagen III and fibronectin in inflammation-linked healing processes rather than fibrotic events, at least at day 4 and 7.

In 1-day-old wild-type and *dy*^*3K*^*/dy*^*3K*^ muscle, collagen III and fibronectin were present at high levels in epimysium (data not shown) and perimysium, whereas their expression in endomysium was less pronounced (especially in case of collagen III) (Fig. 7a,c). Over time, the production of the extracellular matrix components in wild-type muscles was markedly reduced: collagen III was moderately expressed in perimysium (Fig. 7a); fibronectin was weakly expressed in endomysium (day 7, day 14) and mainly confined to blood vessels (day 14, day 21). In contrast, thick deposits of collagen III and fibronectin were present in perimysium of 4-day-old *dy*^*3K*^*/dy*^*3K*^ muscle, and their localisation in 14- and 21-day-old *dy*^*3K*^*/dy*^*3K*^ muscle extended to endomysium (Fig. 7a,c). In 7-day-old *dy*^*3K*^*/dy*^*3K*^ dystrophic muscle in the diseased areas, the expression of collagen III and fibronectin was so robust that it was difficult to delineate the muscle compartments (presumably high expression in perimysium and endomysium).

### Masticatory and respiratory muscles

**Figure 8:**
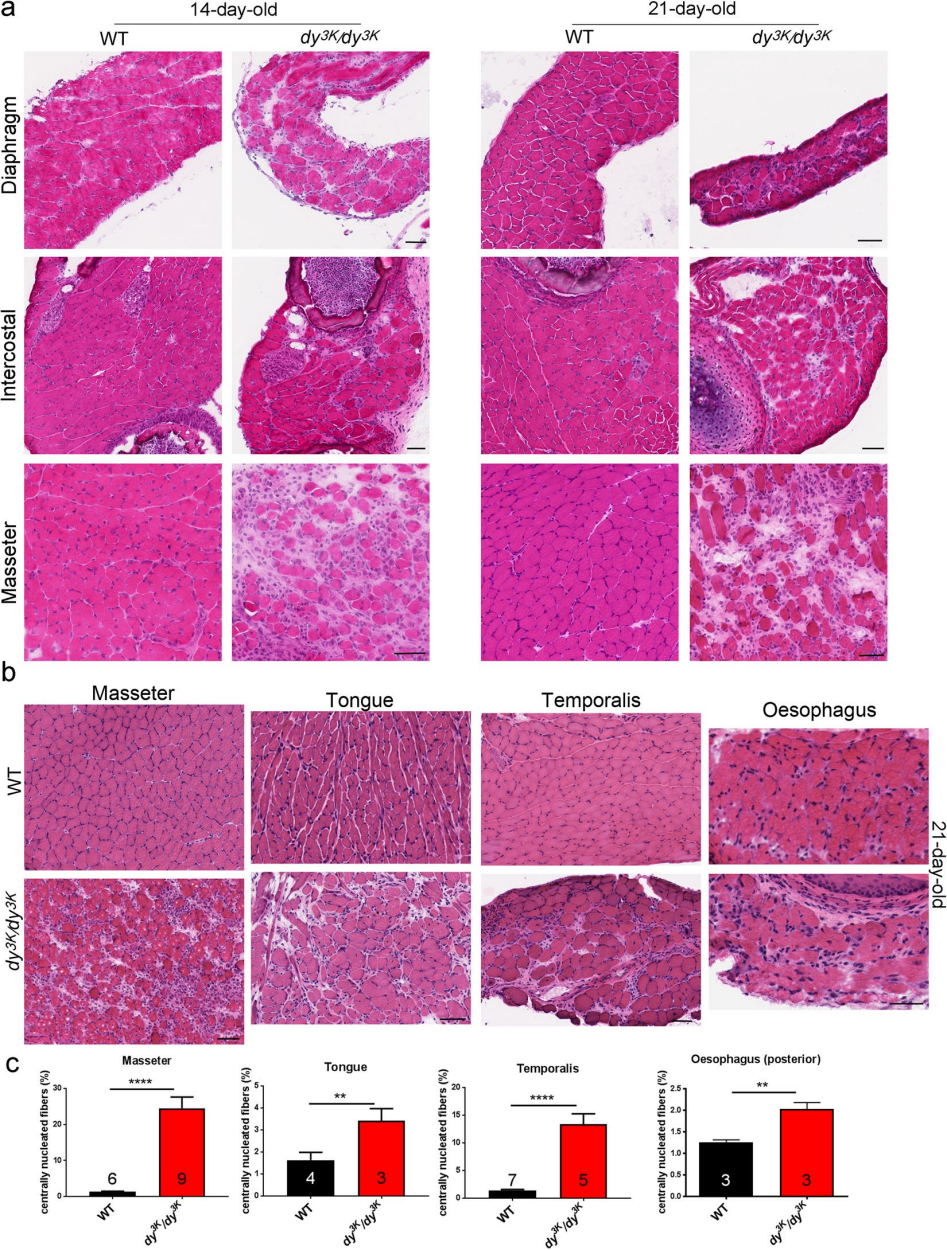
**(a)** Morphology analysis of 14-day-old and 21-day old respiratory muscles (diaphragm, intercostal muscles) and masseter. Diaphragm and intercostal muscles show dystrophic features, but masseter muscle is particularly affected – large areas are devoid of muscle fibres, which are replaced with fibrotic tissue. **(b)** Analysis of various masticatory muscles from 21-day-old wild-type and *dy*^*3K*^*/dy*^*3K*^ mice. Masseter displays the highest degree of deterioration, temporalis and tongue are moderately changed and oesophagus shows very mild dystrophic changes. **(c)** Quantification of centrally nucleated fibres in masticatory muscles. Masseter, tongue, temporalis and oesophagus from *dy*^*3K*^*/dy*^*3K*^ mice show significantly higher amounts of regenerating fibres than corresponding wild-type masticatory muscles (p < 0.0001, p = 0.0334, p < 0.0001, p = 0.0087, respectively). Scale bars: 50 µm.

To date, various muscle types have not been studied in detail in *dy*^*3K*^*/dy*^*3K*^ mice. Since LAMA2-CMD patients suffer from respiratory and feeding complications, we analysed skeletal muscles involved in breathing (diaphragm, intercostal muscles) and feeding (tongue, masseter, temporalis and muscles surrounding oesophagus) in 14-day-old and 21-day-old mice. Respiratory muscles showed a dystrophic pattern at both time points (Fig. 8a). Similar to *dy*^*W*^*/dy*^*W*^ mice^28^, diaphragms from *dy*^*3K*^*/dy*^*3K*^ animals were visibly thinner than wild-type diaphragms (Fig. 8a). They displayed typical hallmarks of dystrophic muscle: central nucleation, infiltration of mononuclear cells and fibrotic lesions (Fig. 8a). Those dystrophic changes ranged from moderate to severe. Internal intercostal muscles showed moderate level of muscular dystrophy (Fig. 8a). However, 21-day-old masticatory *dy*^*3K*^*/dy*^*3K*^ muscles presented varied degrees of dystrophic changes: muscles surrounding oesophagus were either not affected (posterior region, data not shown) or mildly affected (anterior region, Fig. 8b); tongue and temporalis muscle were moderately affected (Fig. 8b), and masseter muscle showed the highest degree of muscle degeneration and muscle wasting (both at day 14 and 21 Fig. 8a). Analysis of central nucleation revealed robust regeneration/degeneration in masseter and temporalis (Fig. 8c) and moderate degenerative process in tongue and oesophagus (Fig. 8c).

### Molecular mechanisms throughout pathology development in *dy*^*3K*^*/dy*^*3K*^ muscle

Dystrophic changes in skeletal muscle stem from a variety of primary and secondary molecular alternations. Several signalling pathways linked to apoptosis, inflammation, metabolism, protein turnover and fibrosis have been shown to be affected in laminin α2 chain-deficient muscle^9–12,29–37^. Our knowledge about regulation of molecular pathways in LAMA2-CMD is limited, and the signalling events identified *in vivo* in mouse models for the disorder have been mainly characterised at the advanced stages of the disease^11,12,29–32,35^. For these reasons, the signalling venue in LAMA2-CMD requires more detailed studies, especially in correlation with different stages of muscle pathology.

**Figure 9:**
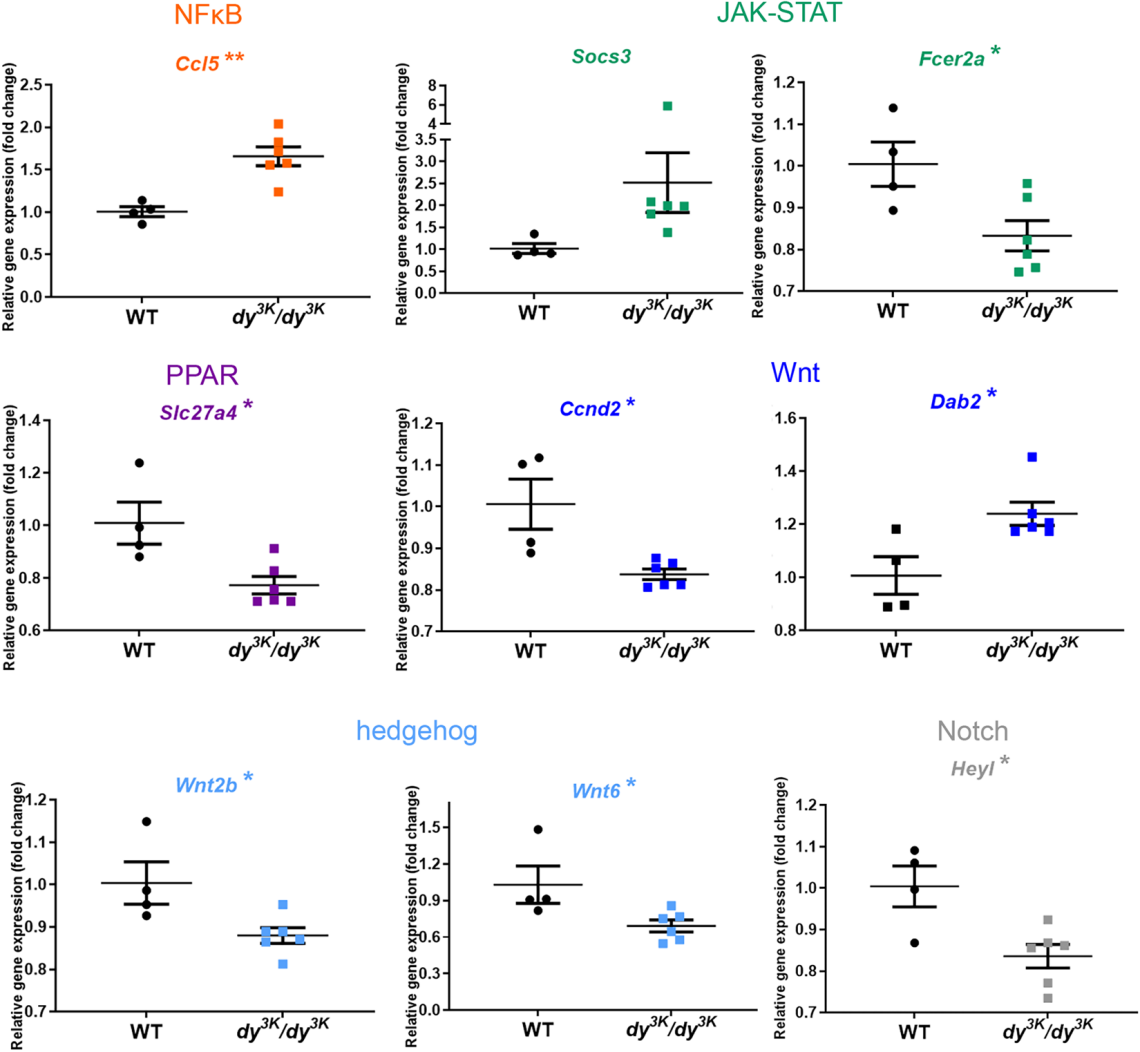
RT^2^ Profiler qPCR analysis of signal transduction pathways target genes in 1-day-old wild-type and *dy*^*3K*^*/dy*^*3K*^ quadriceps. Significantly downregulated and upregulated genes (or genes showing a clear trend for differential expression) are presented. Statistical significance is indicated with asterisks next to the gene abbreviations.

**Figure 10:**
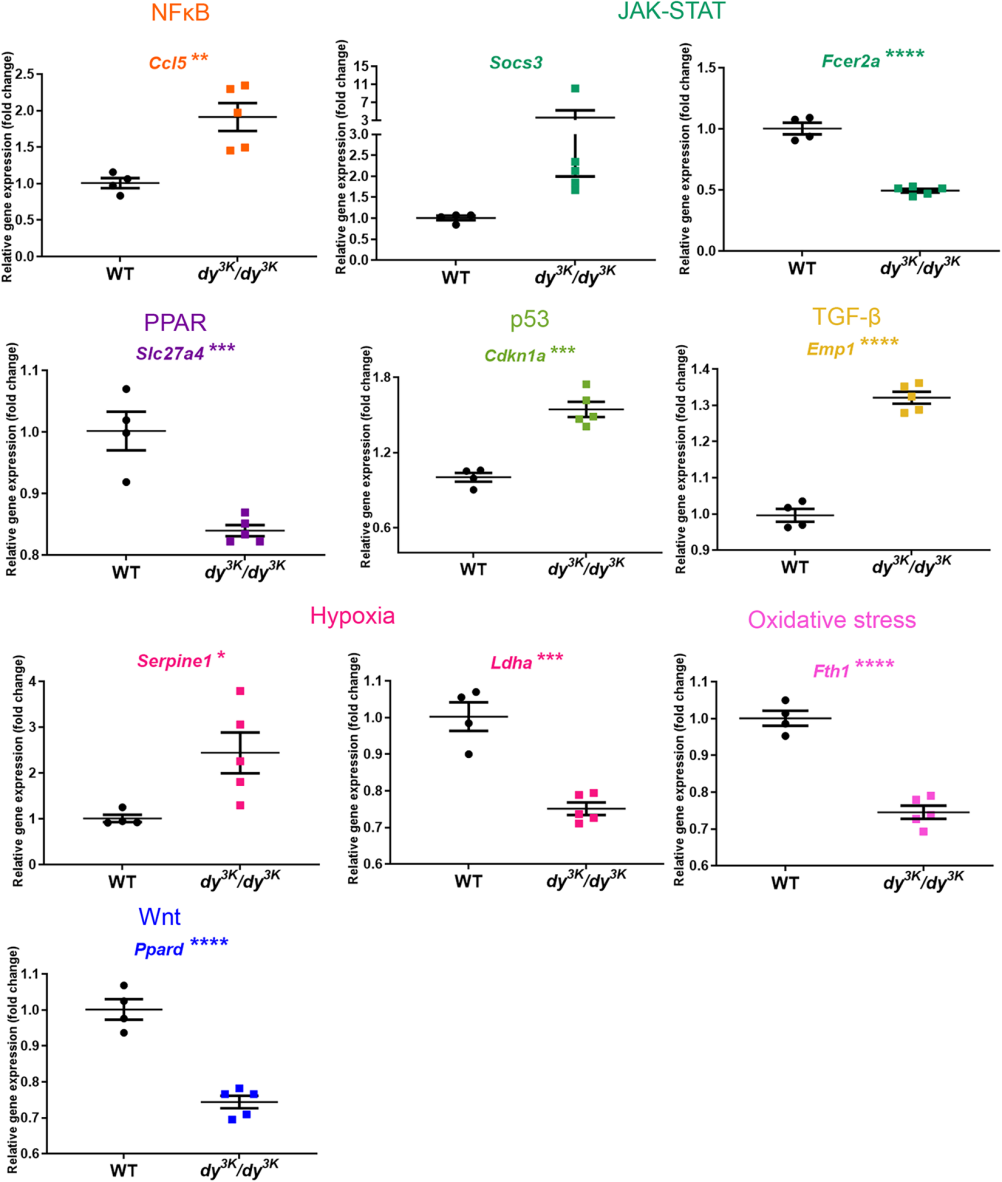
RT^2^ Profiler qPCR analysis of signal transduction pathways target genes in 7-day-old wild-type and *dy*^*3K*^*/dy*^*3K*^ quadriceps. Genes displaying high fold change (including genes showing a clear trend for differential expression) and/or distinct clustering (high statistical significance) are presented. Statistical significance is indicated with asterisks next to the gene abbreviations.

**Figure 11:**
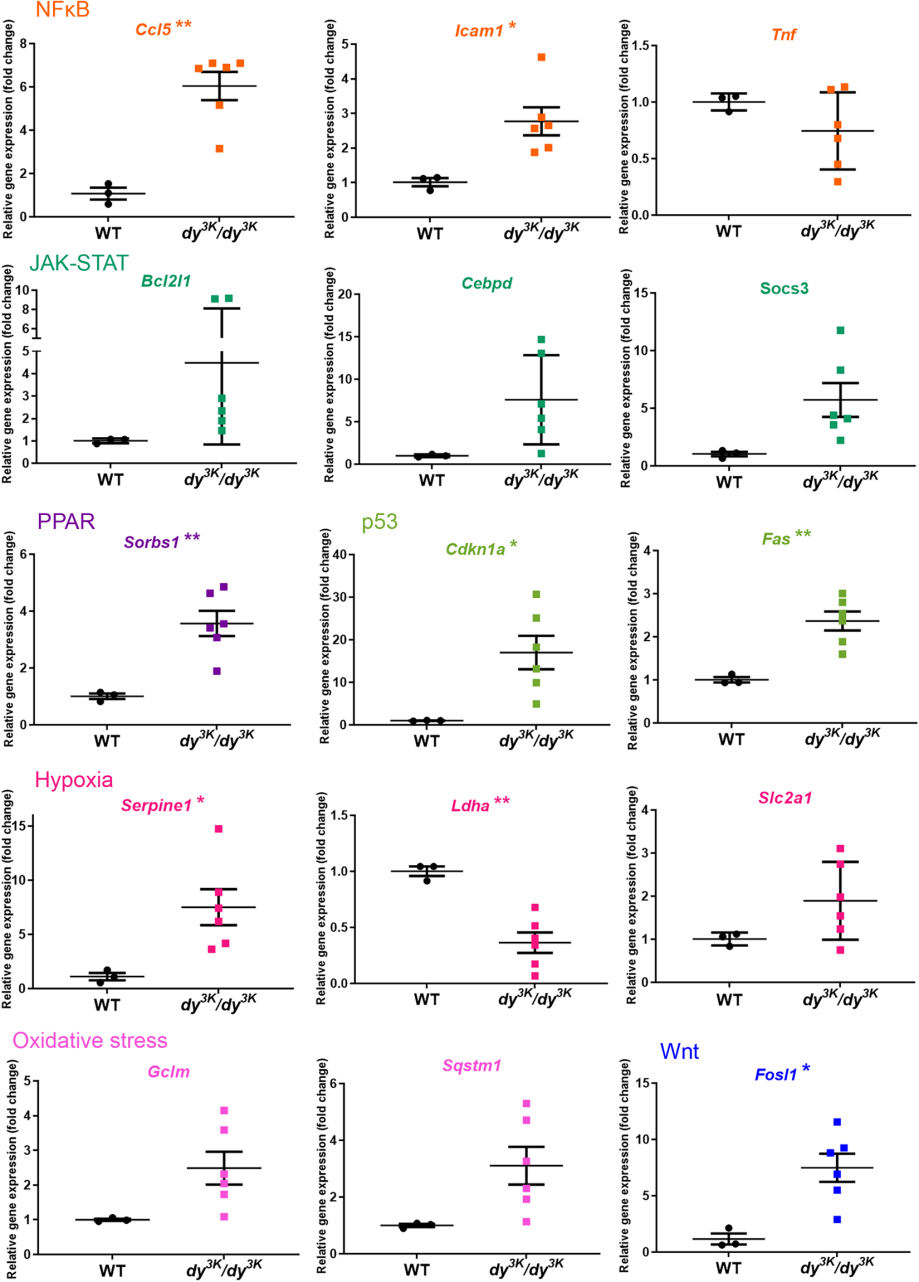
RT^2^ Profiler qPCR analysis of signal transduction pathways target genes in 21-day-old wild-type and *dy*^*3K*^*/dy*^*3K*^ quadriceps. Genes displaying high fold change (including genes showing a clear trend for differential expression) or distinct clustering (high statistical significance) are presented. Statistical significance is indicated with asterisks next to the gene abbreviations.

Very little is known about signalling in *dy*^*3K*^*/dy*^*3K*^ muscle. In order to shed light on molecular events throughout the pathology development in *dy*^*3K*^*/dy*^*3K*^ muscle, we investigated the expression of 84 key target genes responsive to signal transduction activation or inhibition (10 signalling pathways, Signal Transduction PathwayFinder RT^2^ profiler PCR array, Table S1) in 1-, 7- and 21-day-old wild-type and *dy*^*3K*^*/dy*^*3K*^ quadriceps. Genes that showed altered expression already in 1-day-old *dy*^*3K*^*/dy*^*3K*^ muscle were *Ccl5* (NFκB signalling target), *Slc27a4* (PPAR), *Socs3* and *Fcer2a* (JAK-STAT), *Dab2* and *Ccdn2* (Wnt), *Wnt2b* and *Wnt6* (hedgehog), *HeyI* (Notch) (Fig. 9). With pathology progression, additional target genes influenced by other signalling cascades (p53, hypoxia, oxidative stress, TGF-β signalling pathways) were affected in 7-day-old *dy*^*3K*^*/dy*^*3K*^ muscle (Figs 10 and S2). Similarly, a large number of genes (mediated by all the cascades mentioned above) was differentially expressed in 21-day-old *dy*^*3K*^*/dy*^*3K*^ muscle (Figs 11 and S3), suggesting general disruption of muscle homeostasis despite quite efficient regeneration. The genes that showed a high fold change in *dy*^*3K*^*/dy*^*3K*^ muscle at least at one time-point were: *Cdkna1* (p53), *Socs3*, *Cebpd* (JAK-STAT), *Ccl5*, *Icam* (NFκB), *Serpine1* (hypoxia), *Sorbs* (PPAR) and *Fosl1* (Wnt) (Figs 9–11). Differential expression of other genes despite relatively low fold change (Figs 9, S2 and S3), could contribute substantially to pathology development in *dy*^*3K*^*/dy*^*3K*^ muscle.

## Discussion

We have shown that embryonic *dy*^*3K*^*/dy*^*3K*^ muscle appears normal, but already 1-day-old *dy*^*3K*^*/dy*^*3K*^ muscle displays apoptosis. Inflammation becomes evident in 4-day-old *dy*^*3K*^*/dy*^*3K*^ muscle, and muscle damage together with robust inflammation is very pronounced in 7-day-old mice. These pathological events in new-born *dy*^*3K*^*/dy*^*3K*^ mice have not been explored before.

In order to characterise early and late pathology on the molecular level, we screened for differentially expressed signalling transduction gene targets, which steer many cellular processes and which could be powerful mediators of signalling pathways crosstalk. mRNA levels of genes linked to all analysed signalling cascades (NFκB, hedgehog, Wnt, PPAR, JAK-STAT, p53, hypoxia, oxidative stress, TGF-β, and Notch) were affected at least at one time-point in *dy*^*3K*^*/dy*^*3K*^ muscle. This shows an important role for those cascades in propagating dystrophic phenotype of laminin α2 chain-deficiency, but also indicates multidirectional consequences caused by disruption of additional molecules and additional signalling events (domino-like-effect). To our knowledge, gene targets of those commonly analysed pathways have not been studied before in LAMA2-CMD.

While NFκB, JAK-STAT, p53, TGF-β, hypoxia, oxidative stress and PPAR signalling have been directly linked to laminin α2 chain-deficiency, or suggested to be involved in the disease^9,10,29,32,33,36–38^, the contribution of Wnt, hedgehog and Notch cascades to the LAMA2-CMD pathology has not been demonstrated. Interestingly, expression of a few genes driven by these pathways was changed already at day 1 (*Ccnd2*, *Dab2*, *Wnt2b*, *Wnt6* and *HeyI*).

Of the genes that were altered already in 1-day-old quadriceps, expression of *Socs3* and *Ccl5* remained highly increased in *dy*^*3K*^*/dy*^*3K*^ muscle throughout the disease. Inflammation takes a central part in muscle pathology in *dy*^*3K*^*/dy*^*3K*^ mice and cytokines, and growth factors are powerful molecules tipping the scale between homeostasis and pathology. Cytokine expression is de-regulated in *dy*^*3K*^*/dy*^*3K*^ muscle^18^ and Socs3 has a tremendous impact on crosstalk events between signalling pathways that mediate inflammation and fibrosis^39^. Socs3 has therefore gained our attention as a potentially vital regulator of LAMA2-CMD pathology.

Analysis of signalling mediators at the different time points in the *dy*^*3K*^*/dy*^*3K*^ mouse model of laminin α2 chain-deficiency illustrates global disruption of muscle homeostasis already at day 7. Crosstalk between signalling pathways is very complex and one signalling molecule or signal transduction gene targets rarely mediate only one interaction. In order to understand those intricate interactions, a thorough characterisation of signalling molecules is desired. Optimally, RNA sequencing and proteomic studies of embryonic, perinatal and early postnatal laminin α2 chain-deficient muscles should be performed, to (1) pinpoint signalling crosstalks mediating muscle phenotype change, and (2) identify early deregulated genes, proteins and pathways as potential treatment targets.

Because the first dystrophic symptoms in *dy*^*3K*^*/dy*^*3K*^ muscle occur at the early postnatal stages, and the most dramatic pathology is observed between day 7 and 14, we propose that pre-clinical therapy in *dy*^*3K*^*/dy*^*3K*^ mice should start within the first week of life. Most pharmacological attempts in this mouse model have been initiated at the age of 2.5 weeks^11,12,16^ and gave adequate results, but an earlier intervention could be more beneficial for *dy*^*3K*^*/dy*^*3K*^-treated animals. Also, various pharmacological strategies in *dy*^*W*^*/dy*^*W*^ mice have been implemented at the age of 2–3 weeks^28–30,35,40^, when the disease symptoms are already robustly manifested^10^. However, in order to efficiently prevent the muscle pathology of laminin α2 chain-deficiency, we need to decipher the link between inflammation, regeneration, and fibrosis.

It is clear that extracellular matrix remodelling is essential for orchestrated regulation of inflammatory and regenerative events^41–45^. For example, robust production of the extracellular matrix components by muscle stem cells during development and repair is a feature of healthy muscle^41^. Similarly, inflammatory cells are also able to produce matrix molecules^46,47^ and, at the same time, these cells are influenced by changes in the composition of interstitial matrices^45^. Our data suggests that early increase of collagen and fibronectin, as well as acute inflammation, are actually beneficial for the condition of laminin α2 chain-deficient muscle. Targeting certain pathological processes (e.g. inflammation) must therefore be coordinated accordingly throughout the disease progress, to prevent the disruption of muscle healing events. It is not excluded that abolishing inflammation in neonatal *dy*^*3K*^*/dy*^*3K*^ muscle would lead to dramatic deterioration of the dystrophic phenotype^18^. Similarly, aiming at the reduction of the extracellular matrix expression and turnover (TGF-β and CTGF signalling; matrix metalloproteinases and their inhibitors) remains attractive for therapy approaches^48–50^. However, due to the multidirectional impact of the extracellular compartment on muscle regeneration, inflammation and muscle homeostasis, it is essential to learn more about extracellular matrix interactions in diseased muscle.

We have just begun to understand the LAMA2-CMD pathology in more detail. The development of diagnostic techniques (e.g. magnetic resonance imaging, electromyogram, electrical impedance myography, tomography, ultrasound imaging) facilitates relatively non-invasive, detailed characterisation of defects in different tissues of LAMA2-CMD patients^51–53^. For example, these techniques helped to recognise that different muscles from LAMA2-CMD individuals, as in the case of *dy*^*3K*^*/dy*^*3K*^ mice, are affected to various degrees: vastus lateralis, vastus medialis, hamstring group, gluteus, soleus and gastrocnemius show robust atrophy and fatty infiltration, while other muscles (e.g. gracilis, sartorius) show minor involvement in most LAMA2-CMD cases^53–55^. It cannot be excluded that a complicated task of treating entire muscle mass could be narrowed down to fewer muscles, and it needs to be assessed which non-limb muscles that are crucial for feeding and respiration are affected the most. Importantly, magnetic resonance imaging on dystrophic mice to monitor the disease progress and treatment efficiency is now possible^56^.

It is well described that the disease progression in humans is not uniform throughout its course: postnatal worsening is followed by a plateau, temporary improvement and subsequent worsening of the phenotype^57^. It is equally important to characterise the disease progress in mouse models. We conclude that different stages of disease progression could also be observed in *dy*^*3K*^*/dy*^*3K*^ muscle: postnatal worsening (day 4, 7) followed by plateau and phenotype improvement (between day 14–21). Probably due to the early death of *dy*^*3K*^*/dy*^*3K*^ animals, the next wave of muscle deterioration cannot be observed. The time of death coinciding with relatively mild muscle condition of *dy*^*3K*^*/dy*^*3K*^ mice points toward severe defects in other tissues, which have not been fully described.

Necropsy and histopathology findings of *dy*^*3K*^*/dy*^*3K*^ carcasses suggest that the cause of death could be multifactorial, arising from primary muscle defects, mediators released from damaged muscles, pain and distress, undernourishment and hypoventilation and hypoxemia (data not shown). More detailed analysis of organs expressing laminin α2 chain is warranted, in particular lung and intestine. Laminin α2 subunit is expressed both by epithelial and smooth muscle cells in these tissues^58,59^. Pulmonary function is severely impaired in LAMA2-CMD patients, but it has been correlated to weakness of skeletal muscles involved in respiration^7^. It cannot be excluded that the affected individuals have intrinsic lung abnormalities. Patients exhibit feeding difficulties that are attributed to mechanical complications when swallowing^7^ (masticatory/oesophagus muscle weakness), but a possibility of gastrointestinal tract defects has not been addressed. Analysing these tissues in mouse models could pinpoint a potentially important research focus in human patients that is so far unexplored.

Cardiomyocytes and vascular smooth muscle cells are also a rich source of laminin α2 subunit^58,60–62^. Although severe heart involvement has been rarely reported in LAMA2-CMD individuals, left ventricular dysfunction occurs in 30% of cases^6^. Blood vessel defects could have a tremendous impact, not only on the cardiovascular phenotype of LAMA2-CMD patients, but also on the condition of other tissues. In our laboratory, we are currently examining whether vascular smooth muscle and heart function is compromised in the absence of laminin-211. Laminin α2 chain has been shown to be expressed in pancreatic acinar basement membranes^63^ and this could influence glucose balance and general well-being of patients. Finally, many LAMA2-CMD patients suffer from epileptic seizures and display a wide range of brain defects (white matter anomalies such as demyelination and cysts, increased water content in brain, abnormalities of cortical formation (neuron migration abnormalities), posterior fossa malformations, occipital malformations, cerebral hypoplasia)^7,52,57^, yet only blood-brain barrier defect has been clearly described in the *dy*^*3K*^*/dy*^*3K*^ mouse model^64^. Apart from laminin α2 chain presence in brain vessels (astrocyte-derived parenchymal basement membrane)^65,66^, the protein has also been shown to be associated with neurons in various species^67,68^. This could have repercussions on neuronal function in laminin α2 chain-deficient murine brain. Greater attention should therefore be given to the non-skeletal muscle manifestations in mouse models for LAMA-CMD.

To summarise, a comprehensive picture of LAMA2-CMD pathogenesis is indispensable for designing efficient preclinical studies and future clinical trials. Our data is one of the puzzle pieces that significantly contributes to understanding of the laminin α2 chain-deficiency.

## Materials and Methods

### Mice

Laminin α2 chain-deficient *dy*^*3K*^*/dy*^*3K*^ mice^69^ were compared to wild-type littermates. Mice were maintained and bred in the animal facilities of Biomedical Center (Lund). All experimental procedures involving animals were approved by the Malmö/Lund (Sweden) Ethical Committee for Animal Research (ethical permit number: M152-14 and M8173-19) in accordance with the guidelines issued by the Swedish Board of Agriculture.

### Functional tests

Two-, 7- and 11-day-old mice (*dy*^*3K*^*/dy*^*3K*^ and healthy controls) were subjected to righting reflex test and hind limb suspension test. The tests were performed according to the standardised operating procedures recommended by Treat-NMD network (protocol MD_M.2.2.002 and SMA_M.2.2.001, respectively)^21^. In the righting test, a mouse was placed on an even surface on its back and stabilised with a finger so that all four paws were pointing upwards. Once the finger was removed, time was measured (7- and 11-day-old mice) until the mouse turned itself on its belly and spread all paws on the bench (for max 60 seconds). It was taken into consideration when a paw/paws remained non-stretched under the trunk after 60 seconds (despite trunk righting) (see results). For 2-day-old mice, instead of exact time measurements, a scoring system was applied: 0: no attempt to right itself, very passive; 1: poor attempt to right itself; 2: fair attempt to right itself, active; 3: managed to turn. Each mouse was tested three times, with resting time in between trials when the mice were placed on a heating pad (37 °C). Average time/score was taken from the three attempts.

For the hind limb suspension test, a mouse was placed facing downwards inside a 50 ml conical tube, with its hind legs over the rim of the tube. Time was measured until the mouse fell from the rim. Three consecutive trials were performed with resting time in between trials. The hind limb suspension score was estimated during the first 10–15 seconds of the test: the degree of limb spreading on the rim, ranging from 0 (constant clasping of the hind limbs, low tail) to 4 (full spreading of paws, no apparent weakness)^21^.

Eleven and 14-day-old mice were subjected to the hang-wire grip/suspension test^20,22,70^ and 14-day-old mice were subjected to the mesh grip test^19,20,23^ (both tests described in Treat-NMD standard operating procedures, protocol SMA_M.2.1.002). In the hang-wire grip test, mice were held by the tail and allowed to grasp a wire suspended in mid-air. Animals were released when they displayed a stable grip with both forelimbs. The time a mouse managed to hold onto the wire was measured. Three trials were performed with a rest period between trials. In the mesh grip test, each pup was placed on a wire mesh (0.25 cm^2^). The mesh was slowly inverted. If the mouse held onto the mesh when inverted to 180 degrees, the latency to fall was recorded. The test was repeated three times with resting time in between trials.

Eleven and 14-day-old mice were subjected to the tail suspension test (reflecting hind limb self-clasping)^21^. Animals were suspended by the tail for 15 seconds. Their hind limb position was scored from 0–4 (4: hind limbs spread open; 3: hind limbs not completely spread; 2: limbs often together; 1: limbs often close together, occasionally clasped; 0: limbs always close together, often clasping). The test was repeated three times.

An open field activity test^14^ (time spent on active exploration of a new cage for 5 minutes) was also performed on 14-day-old animals.

Mice were sacrificed and genotyped after functional tests (longitudinal analysis of each individual was not possible). Tests on 7-day-old and older mice were not blinded, due to the obvious phenotype of *dy*^*3K*^*/dy*^*3K*^ pups.

### Histology

Quadriceps femoris and calf muscles were isolated from 18.5-day-old embryos and from 1-, 4-, 7, 14- and 21-day-old mice (wild-type and *dy*^*3K*^*/dy*^*3K*^); masseter, diaphragm and intercostal muscles were isolated from 14- and 21-day-old mice; tongue, temporalis and oesophagus were isolated from 21-day-old animals. Tissues were embedded in OCT (Tissue Tek) and frozen in liquid nitrogen. Cryo-sections (cross-sections, 7 µm thick) were stained with hematoxylin and eosin^13^, scanned using Aperio’s Scanscope CS2 (with Scanscope console v.8.2.0.1263), and images were created using Aperio software.

### Immunofluorescence

Muscle cryo-sections (cross-sections, from all age groups) were processed for immunofluorescence experiments following standard procedures. Primary antibodies against caspase-3, CD11b, CD68, collagen III and fibronectin were used^13^. Biotinylated wheat germ agglutinin (WGA) was detected with fluorescein avidin D (Vector Laboratories, Burlingame, CA). Quadriceps (rectus femoris) was used for immunolabelling with CD11b, collagen III and fibronectin antibodies, whereas calf muscles and quadriceps were used for caspase-3 staining. Sections were analysed and images were captured using Zeiss Axioplan fluorescence microscope, ORCA 1394 ER digital camera, and Openlab 4 software.

### Morphometry and fluorescence quantification

Whole rectus femoris was considered for morphometry and immunofluorescence quantifications. The number of fibres and the number of fibres with centrally located nuclei was assessed using ImageJ software version 1.43 u, Cell Counter plug-in (NIH). The cross-sectional area of WGA-stained muscle fibres and the area of the whole muscle (stitched photos) were measured using Adobe Photoshop CS5 extended version (Adobe Systems, San Jose, CA). Muscle fibre diameter was measured in Image J to verify the cross-sectional area results (data not shown).

For quantification of CD11b and collagen III immunostaining, multiple Tiff-format images of rectus femoris cross sections were taken at 10x magnification and stitched using Adobe Photoshop software. The quantification was performed using ImageJ software. The area corresponding to CD11b or collagen III labelling was quantified relative to the entire area of the rectus femoris cross section. The measurements were set to a threshold that was manually adjusted for each individual image (the total muscle area versus stained area, measured in square pixels).

### RNA isolation and RT^2^ Profiler PCR Array

RNA was isolated from 1-, 7- and 21-day-old quadriceps muscles using Qiagen RNeasy Plus Universal Kit (Qiagen), following the manufacturer’s specifications. The quality and concentration of RNA were assessed using Agilent 2100 Bioanalyzer (Agilent RNA 6000 Nano Kit). One microgram of muscle RNA was used to synthesise cDNA (RT^2^ first strand Kit, Qiagen). Genomic DNA elimination was performed prior to the reverse transcription (RT^2^ first strand Kit, Qiagen). Mouse Signal Transduction PathwayFinder RT^2^ Profiler PCR Array (Qiagen) was used for wild-type and *dy*^*3K*^*/dy*^*3K*^ cDNA samples, according to the manufacturer’s specifications. Briefly, RT^2^ SYBR Green PCR mastermix was mixed with each cDNA synthesis reaction and 25 μl were distributed into each well of an individual 96 well-RT^2^ Profiler PCR plate (each well containing primers for one specific gene). The amplification was performed in a Light Cycler 480 Real-Time PCR System (Roche). Five reference genes, genomic DNA control, reverse transcription control and positive PCR control wells were included in each array. Hsp90 was chosen as a reference gene (the C_p_ values did not differ between genotypes throughout the three time points). All controls mentioned above passed the quality requirements specified by the manufacturer. Comparative CT method was used for relative quantitation. A few genes were excluded from analysis due to a presence of unspecific products (two peaks in melting curves) or C_p_ value above 35 (considered a negative call).

### Statistics

Statistical analyses were performed using GraphPad Prism 7 software. Unpaired student’s t-test was used to determine differences between two groups (wild-type group versus age-matched *dy*^*3K*^*/dy*^*3K*^ group). Statistical significance was accepted for *P* < 0.05. Averaged data were reported as means ± SEM. The numbers of animals used in each group are indicated in graph bars or figure legends.

## Supplementary information

Supplemetary Figures and Table

Video 1

Video 2

## Data Availability

The datasets generated during and/or analysed during the current study are available from the corresponding author on reasonable request.
