# Supplementary material for: Early skeletal muscle pathology and disease progress in the dy3K/dy3K mouse model of congenital muscular dystrophy with laminin α2 chain-deficiency
Source: Sci Rep. 2019 Oct 4;9:14324. doi: 10.1038/s41598-019-50550-0 (PMC6778073; doi:10.1038/s41598-019-50550-0)
Supplement: Supplementary file 1 — Supplemetary Figures and Table [file 41598_2019_50550_MOESM1_ESM.docx]

**Early skeletal muscle pathology and disease progress in the *dy^3K^/dy^3K^* mouse model of congenital muscular dystrophy with laminin α2 chain-deficiency**

Kinga I. Gawlik*^§^, Zandra Körner, Bruno M. Oliveira^†^, Madeleine Durbeej^§^

Muscle Biology Unit, Department of Experimental Medical Science, Lund University, Lund, Sweden

^§^ Shared last authorship

^†^ Deceased

*Corresponding author:

Kinga I. Gawlik

Muscle Biology Unit

Department of Experimental Medical Science

BMC C12, Lund University

221 84 Lund, Sweden

Tel : 0046 46 2220813

Fax: 0046 46 2220855

Email: [kinga.gawlik@med.lu.se](mailto:kinga.gawlik@med.lu.se)

| Gene | Pathway |
| --- | --- |
| *Acsl3* | PPAR signalling |
| *Acsl4* | PPAR signalling |
| *Acsl5* | PPAR signalling |
| *Adm* | Hypoxia signalling |
| *Arnt* | Hypoxia signalling |
| *Atf4* | TGF-β signalling |
| *Axin2* | Wnt signalling |
| *Bax* | p53 signalling |
| *Bbc3* | p53 signalling |
| *Bcl2* | Hedgehog signalling |
| *Bcl2a1a* | NFκB signalling |
| *Bcl2l1* | JAK-STAT signalling (STAT3 and STAT5-induced), NFκB signalling |
| *Bir3* | NFκB signalling |
| *Bmp2* | Hedgehog signalling |
| *Bmp4* | Hedgehog signalling |
| *Btg2* | p53 signalling |
| *Car9* | Hypoxia signalling |
| *Ccl5* | NFκB signalling |
| *Ccnd1* | Wnt signalling, JAK-STAT signalling (STAT3 and STAT5-induced) |
| *Ccnd2* | Wnt signalling |
| *Cdkn1a* | p53 signalling |
| *Cdkn1b* | TGF-β signalling |
| *Cebpd* | JAK-STAT signalling (STAT3-induced) |
| *Cpt2* | PPAR signalling |
| *Csf1* | NFκB signalling |
| *Dab2* | Wnt signalling |
| *Egfr* | p53 signalling |
| *Emp1* | TGF-β signalling |
| *Epo* | Hypoxia signalling |
| *Fabp1* | PPAR signalling |
| *Fas* | p53 signalling |
| *Fcer2a* | JAK-STAT signalling (JAK1 & JAK3/STAT6-induced) |
| *Fosl1* | Wnt signalling |
| *Fth1* | Oxidative stress |
| *Gadd45a* | p53 signalling |
| *Gadd45b* | TGF-β signalling |
| *Gata3* | JAK-STAT signalling (JAK1 & JAK3/STAT6-induced) |
| *Gclc* | Oxidative stress |
| *Gclm* | Oxidative stress |
| *Gsr* | Oxidative stress |
| *Herpud1* | TGF-β signalling |
| *Hes1* | Notch signalling |
| *Hes5* | Notch signalling |
| *Hey1* | Notch signalling |
| *Hey2* | Notch signalling |
| *HeyI* | Notch signalling |
| *Hmox* | Hypoxia signalling, Oxidative stress |
| *Icam* | NFκB signalling |
| *Id1* | Notch signalling |
| *Ifng* | NFκB signalling |
| *Ifrd1* | TGF-β signalling |
| *Irf1* | JAK-STAT signalling (JAK1 & JAK2/STAT1-induced) |
| *Jag1* | Notch signalling |
| *Ldha* | Hypoxia signalling |
| *Lfng* | Notch signalling |
| *Lrg1* | JAK-STAT signalling (STAT3-induced) |
| *Mcl1* | JAK-STAT signalling (STAT3-induced) |
| *Mmp7* | Wnt signalling |
| *Myc* | TGF-β signalling, Wnt signalling |
| *Notch1* | Notch signalling |
| *Nqo1* | Oxidative stress |
| *Olr1* | PPAR signalling |
| *Pcna* | p53 signalling |
| *Ppard* | Wnt signalling |
| *Ptch1* | Hedgehog signalling |
| *Rb1* | p53 signalling |
| *Serpine1* | Hypoxia signalling |
| *Slc27a4* | PPAR signalling |
| *Slc2a1* | Hypoxia signalling |
| *Socs3* | JAK-STAT signalling (STAT3 and STAT5-induced) |
| *Sorbs1* | PPAR signalling |
| *Sqstm1* | Oxidative stress |
| *Stat1* | NFκB signalling |
| *Tnf* | NFκB signalling |
| *Tnfsf10* | TGF-β signalling |
| *Txn1* | Oxidative stress |
| *Txnrd1* | Oxidative stress |
| *Vegfa* | Hypoxia signalling |
| *Wisp1* | Wnt signalling |
| *Wnt1* | Hedgehog signalling |
| *Wnt2b* | Hedgehog signalling |
| *Wnt3a* | Hedgehog signalling |
| *Wnt5a* | Hedgehog signalling |
| *Wnt6* | Hedgehog signalling |
| *Actb* | Reference gene |
| *B2m* | Reference gene |
| *Gapdh* | Reference gene |
| *Gusb* | Reference gene |
| *Hsp90ab1* | Reference gene |

**Supplementary Table S1.** Panel of genes included in RT^2^ Profiler qPCR analysis. Genes marked in red were excluded from analysis.


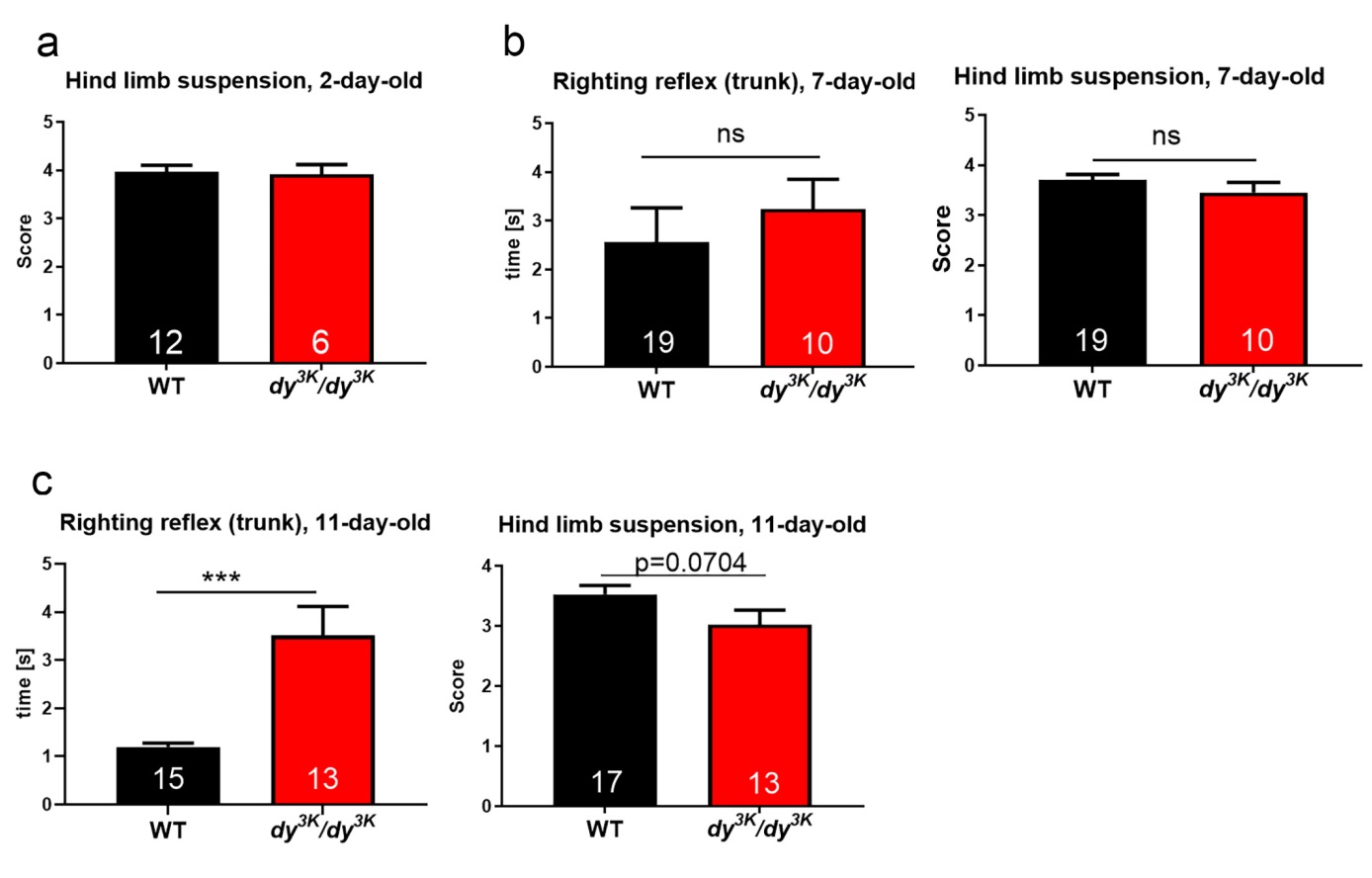


**Supplementary Figure S1.** Muscle function in *dy^3K^/dy^3K^* mice throughout the disease course. (a) Hind limb suspension test (score) in 2-day-old mice. (b, c) Righting reflex (not taking into account whether all limbs spread onto the surface, only trunk change of position was considered) and hind limb suspension test (score) in 7- and 11-day-old animals. The degree of limb spreading on the rim during hind limb suspension test was scored as follows: 0 (constant clasping of the hind limbs, low tail) to 4 (full spreading of paws, no apparent weakness).

**
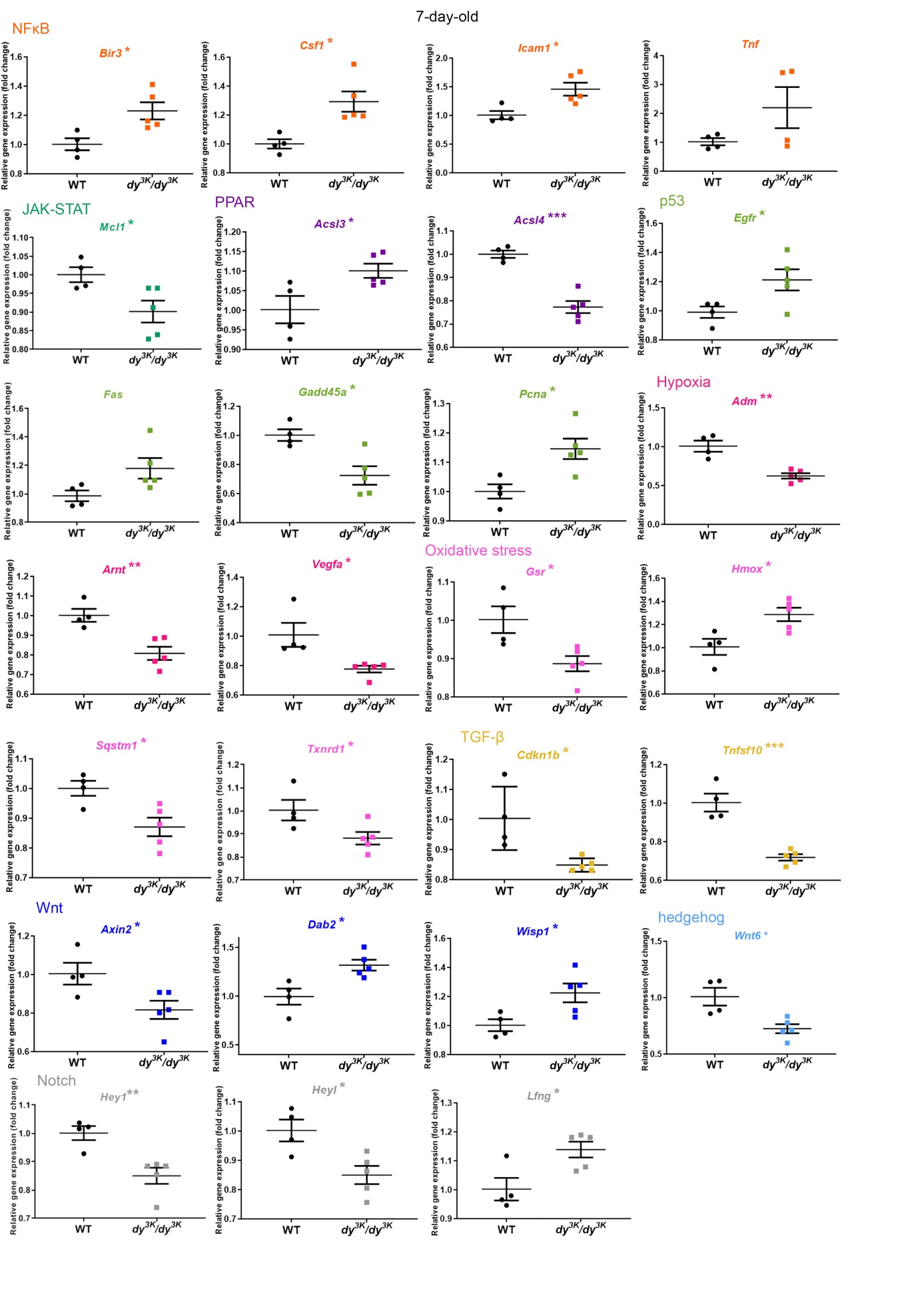
Supplementary Figure S2.** RT^2^ Profiler qPCR analysis: differentially expressed genes showing relatively low fold change in 7-day-old *dy^3K^/dy^3K^* muscle compared with gene expression in age-matched wild-type muscle.

**
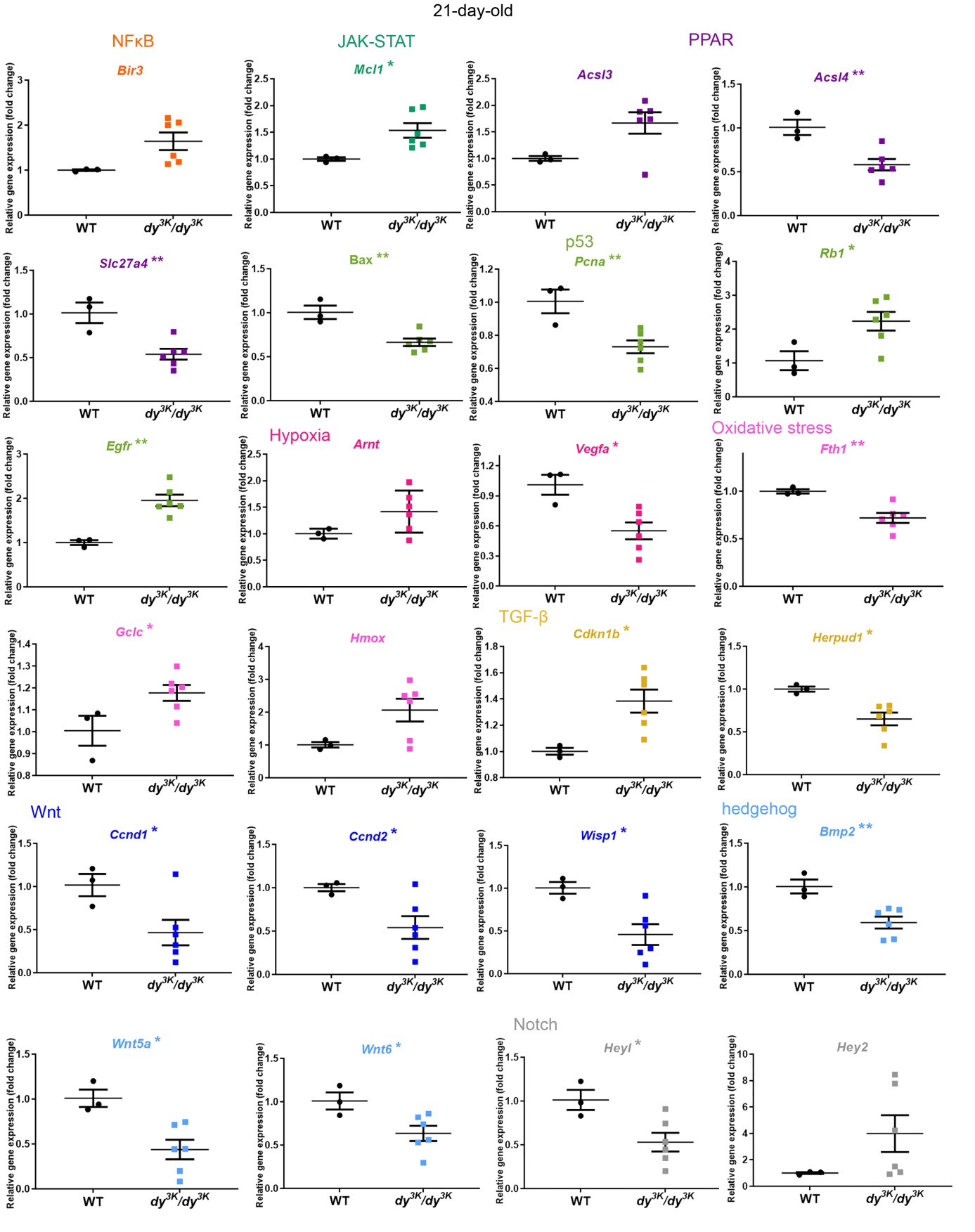
Supplementary Figure S3.** RT^2^ Profiler qPCR analysis: differentially expressed genes showing relatively low fold change in 21-day-old *dy^3K^/dy^3K^* muscle compared with gene expression in age-matched wild-type muscle.

**Video S1.** Fourteen-day-old wild-type mouse performing the hang-wire suspension test.

**Video S2.** Fourteen-day-old *dy^3K^/dy^3K^* mouse struggling during the hang-wire suspension test.
